# Catecholamine crisis after endoscopic ultrasound-guided fine-needle biopsy: A case report

**DOI:** 10.1097/MD.0000000000032458

**Published:** 2022-12-23

**Authors:** Hyun Seok Lee, Eaum-Seok Lee

**Affiliations:** a Division of Gastroenterology, Department of Internal Medicine, Chungnam University Hospital, Daejeon, Korea.

**Keywords:** catecholamines, endoscopic ultrasound, guided fine, needle aspiration

## Abstract

The function and use of endoscopic ultrasound-guided fine-needle biopsy (EUS-FNB) has developed in recent years, particularly in distinguishing malignant from benign masses. It is a generally safe method that can be completed without any uncommon complications. Here, although no complications from EUS-FNB were noted, we report about an unexpected catecholamine crisis that occurred after EUS-FNB.

**Patient’s concerns:**

A 60-year-old man visited an outpatient clinic with an incidental diagnosis of a retroperitoneal mass on his last abdominopelvic computed tomography (APCT) scan taken at the time of a previous liver abscess treatment. On presentation, the patient showed no symptoms.

**Diagnoses:**

A retroperitoneal mass was incidentally discovered on APCT, and risk for lymphoma, gastrointestinal tumor, or neuroendocrine tumor was noted on the APCT reading.

**Interventions:**

EUS-FNB was performed on retroperitoneal mass.

**Outcomes:**

The procedure was completed without any complications; however, the patient’s condition deteriorated due to hemodynamic instability and cardiovascular collapse. During intensive care unit (ICU) treatment, the biopsy results were found to be paraganglioma. Catecholamine crisis occurred after biopsy of paraganglioma.

**Lessons:**

The case presented here gives a caution of complication that may occur after EUS-FNB. Although EUS-FNB is known to be relatively safe, careful evaluation is required when performing biopsy of lesions around the aorta.

## 1. Introduction

Primary retroperitoneal mass refers to a rare but diverse neoplasm and non-neoplasm that occurs within the retroperitoneal space but separately from the retroperitoneal organ.^[1]^ Among retroperitoneal masses, solid neoplastic mass is typically lymphoma, sarcoma, and neurogenic tumors, and solid nonneoplastic mass is retroperitoneal fibrosis.^[2]^ Retroperitoneal mass is mainly diagnosed using APCT or magnetic resonance imaging, and approximately 75% of retroperitoneal mass is known to be malignant.^[3]^ Therefore, histological evaluation is crucial to distinguish malignancy, and recently, the usefulness of EUS-FNB has been proven, and this method has been mainly used as a biopsy method.^[4]^

We report a case of catecholamine crisis, an unexpected complication after EUS-FNB with a retroperitoneal mass. Careful differentiation of retroperitoneal mass before the procedure may be required, and in particular, a prior evaluation of catecholamine-producing tumor may be required.

This case report was approved by the Institutional Review Board of Chungnam National University Hospital. The patient provided consent for publication of this report.

## 2. Case report

A 60-year-old man visited an outpatient clinic on August 17, 2021, concerning a mass on an APCT scan confirmed 2 months ago from when the patient visited the emergency room for a fever that persisted for 15 days. Here, he was treated for his liver abscess by receiving percutaneous catheter drain insertion and antibiotic treatment. A 5-cm-sized retroperitoneal mass was accidentally found along with a liver abscess in APCT taken in the emergency room, and after liver abscess treatment, he visited an outpatient clinic for evaluation of the retroperitoneal mass.

At the time of his outpatient visit, the abscess was completely cured, and there were no symptoms of the disease such as headache, sweating, and palpations. The patient has a history of hypertension, diabetes, and hyperlipidemia and takes antihypertensives, oral hypoglycemic agents, insulin, and statins. His blood pressure is well controlled. The patient drank 5 bottles of soju every day for 30 years and does not smoke. Family history is unremarkable.

During his outpatient visit, the patient was admitted for EUS-FNB on August 17, 2021. At the time of admission, vital signs were within range. The complete blood count, chemistry, and tumor markers were tested and revealed no pathologies.

**Figure 1.**
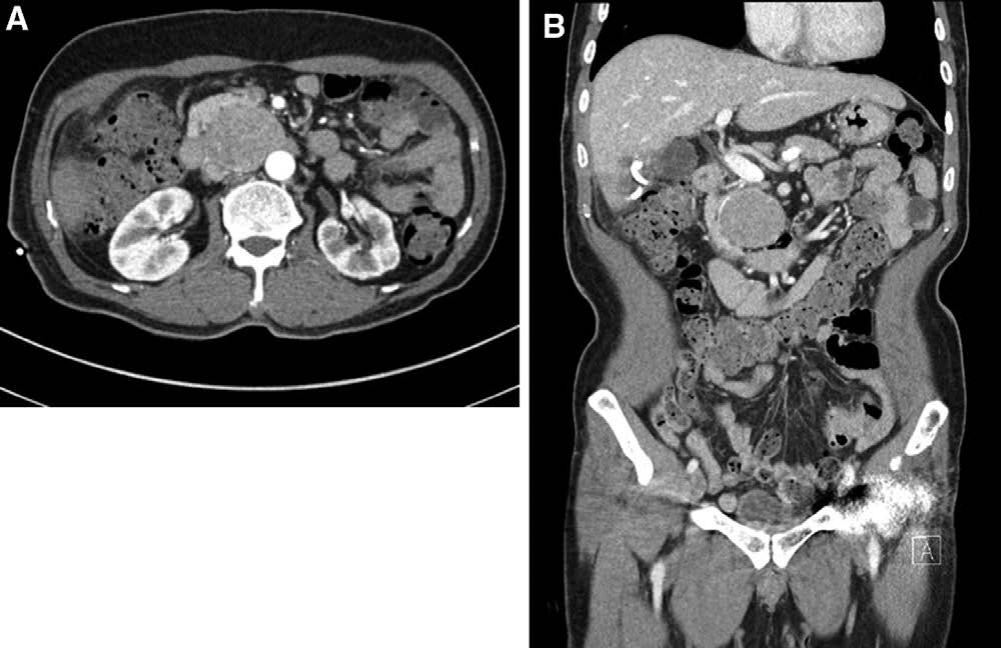
Abdominal CT taken before hospitalization. A solid mass of approximately 5 cm in size is observed in the aortocaval area. (1A) Axial plane and (1B) coronal plane.

Since the last APCT scan was taken on June 29, 2021, a follow-up APCT scan was performed. The new scan revealed that the liver abscess was cured and the size of the retroperitoneal mass remained unchanged at 5 cm. No other pathologies were found. The APCT report showed a differential of lymphoma, gastrointestinal stromal tumor, and neuroendocrine tumor (Fig. 1).

**Figure 2.**
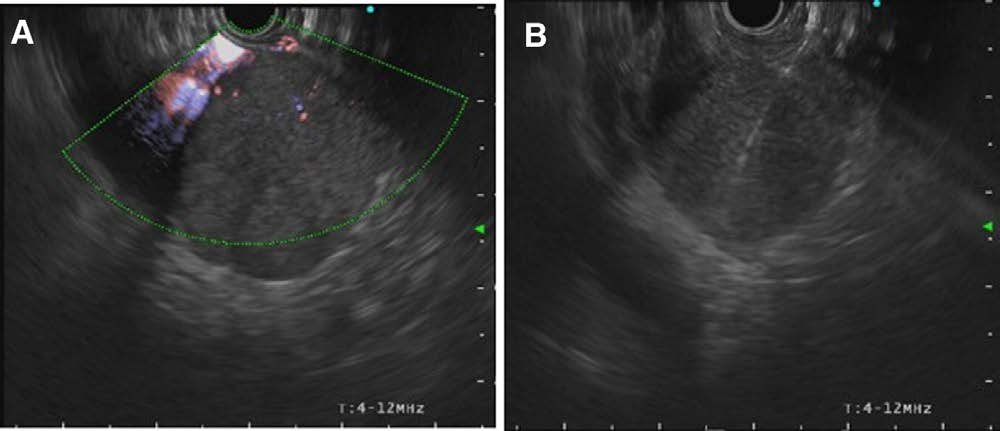
EUS-FNB photo. A relatively well-defined mass of approximately 5 cm in size in the aortocaval space was biopsied using a 22-gauge needle. (2A) Vascularity was confirmed on doppler setting and (2B) needling with a 22-gauge needle. EUS-FNB = endoscopic ultrasound-guided fine-needle biopsy

The EUS-FNB was performed on August 18, 2021, the day after his hospitalization. On EUS, the mass was assessed to be 5 cm in size, well-defined, and located in the aortocaval space. A tissue sample of the mass was collected using the fanning method with a 22-gauge needle after 3 attempts (Fig. 2). The procedure was completed without any specificity.

Approximately an hour after the procedure, the patient complained of sweating, dyspnea, and nausea. His vital signs were as follows: blood pressure, 189/132 mm Hg; heart rate, 102 beats/min; respiratory rate, 24 breathes/min; and oxygen saturation, 74%. Eventually, endotracheal intubation was required as the oxygen saturation worsened despite oxygen therapy. On intubation, a large amount of mucous was found in the airways. A chest PA taken after the intubation revealed increased infiltration in both lung fields. The patient was then moved to the intensive care unit (ICU).

**Figure 3.**
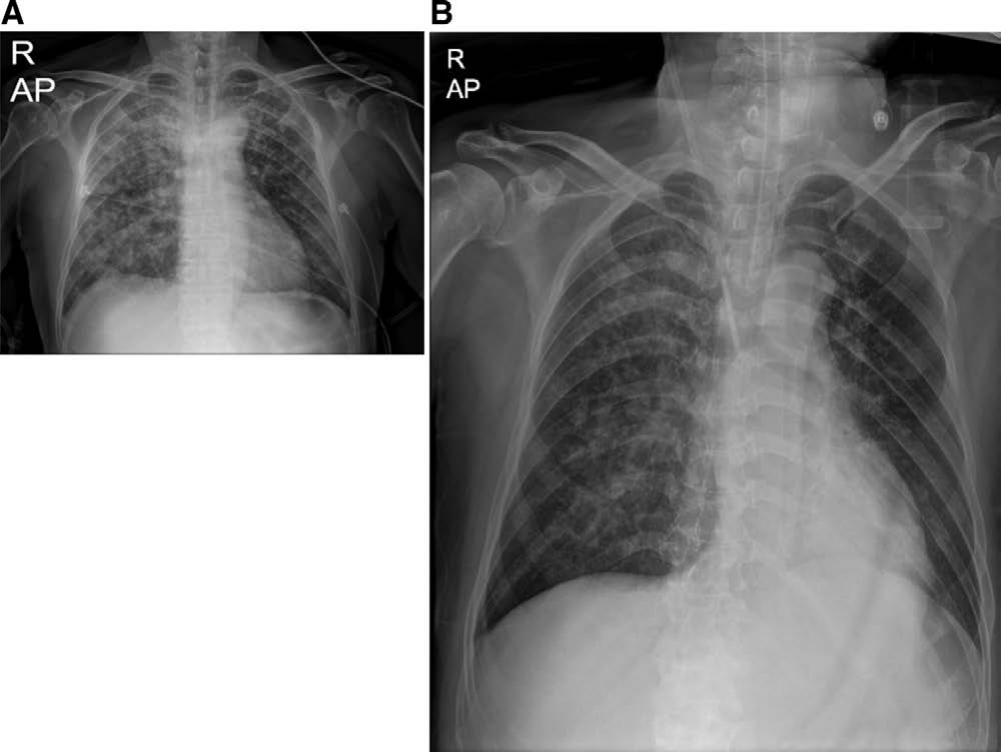
Chest PA before and after hemodialysis. Both lung infiltration improved after dialysis compared with that before dialysis. (3A) Increased infiltration in both the lungs before hemodialysis. (3B) Improved infiltration in both the lungs after hemodialysis.

In the ICU, the patient experienced a repeated pattern of sinus tachycardia with a heart rate ≥ 160 beats per min when his blood pressure rose to ≥ 180/130 mm Hg and a drop in the heart rate as the blood pressure dropped to 80/60 mm Hg. To counteract the hemodynamic instability, a vasopressor and inotropics such as norepinephrine, vasopressin, and dobutamine were administered. Esmolol, a beta-blocker, was administered for heart rate control. An echocardiography in the ICU confirmed stress-induced cardiomyopathy in the patient. On the next day, a drop in renal function and urine output was noticed, and the patient was put on hemodialysis for 5 days. After the hemodialysis, a chest PA showed improvement of lung infiltration and revealed underlying pulmonary edema (Fig. 3).

**Figure 4.**
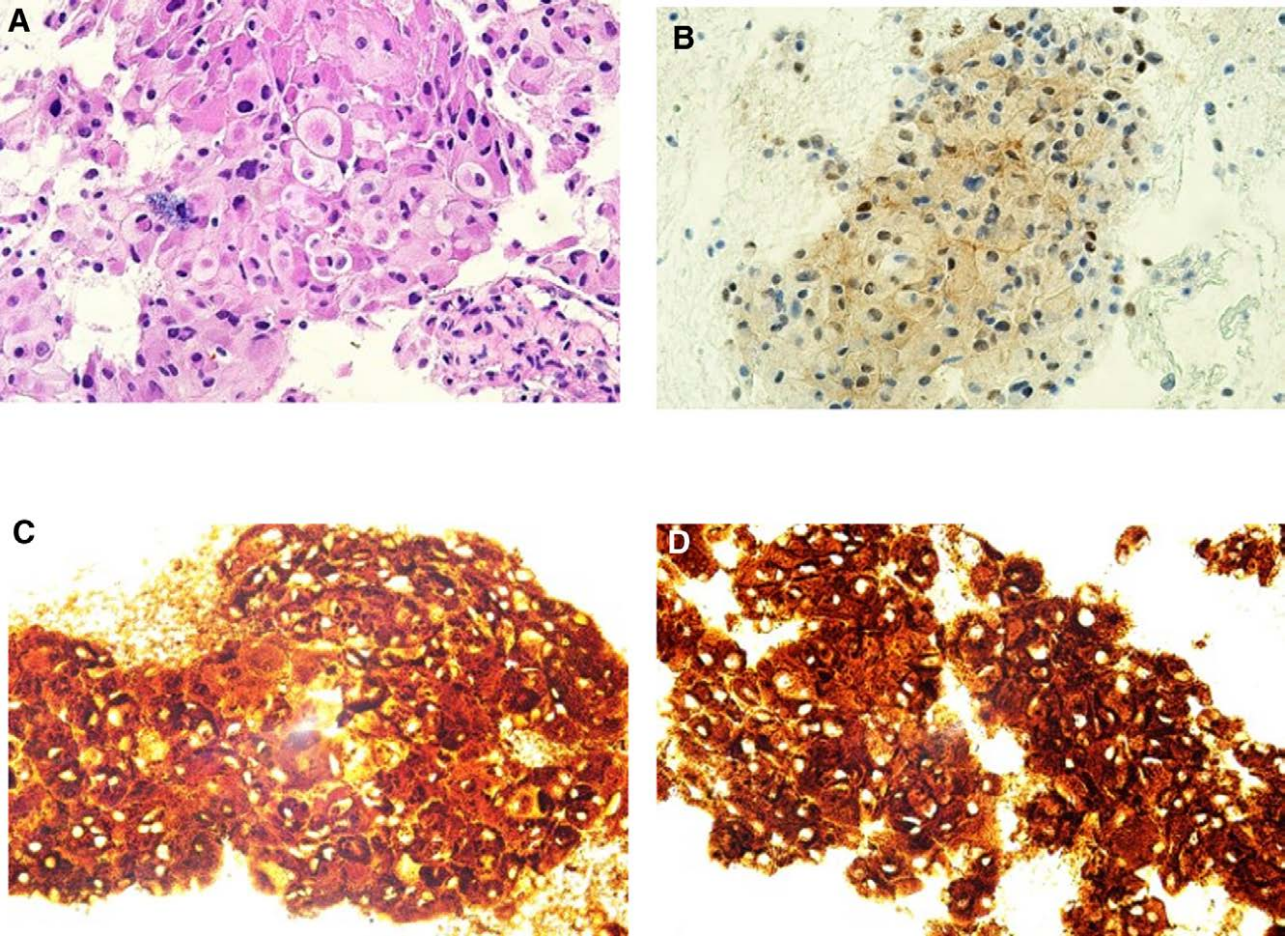
Histopathology photo. (4A) Nested architecture and distinct Zellballen pattern separated by fibrovascular stroma and formed by epithelioid cells (H&E, ×400). Immunohistochemical staining showing diffuse positivity for (4B) S-100 (×400), (4C) chromogranin (×400), and (4D) synaptophysin (×400).

After 1 week of treatment in the ICU, EUS-FNB results arrived. Immunohistochemical staining showed positivity for S-100, chromogranin, and synaptophysin, and the findings suggested paraganglioma (Fig. 4). Despite histological confirmation, catecholamines were further evaluated in blood and urine tests. Plasma results revealed metanephrine > 20.21 (0 < normal < 0.49 nmol/L) and normetanephrine > 45.33 (0 < normal < 0.89 nmol/L). Urine tests showed the following: metanephrine > 1814.7 (52 < normal < 341 µg/day); normetanephrine > 3814.3 (88 < normal < 444 µg/day); epinephrine, 83.3 (0 < normal < 20.0 µg/day); norepinephrine, 398.0 (15.0 < normal < 80.0 µg/day); and vanillylmandelic acid, 22.0 (0 < normal < 8 mg/day) (Table 1).

**Table 1:** Catecholamine test results of serum and urine.

**Plasma**		
Metanephrine	>20.21	0–0.49 nmol/L
Normetanephrine	>45.33	0–0.89 nmol/L
**Urine**		
Metanephrine	>1814.7	52–341 µg/d
Normetanephrine	>3814.3	88–444 µg/d
Epinephrine	83.3	0.0–20.0 µg/d
Norepinephrine	398.0	15.0–80.0 µg/d
VMA*	22.0	0–8 mg/d

* Vanillylmandelic acid.

The patient’s symptoms could be explained only after the diagnosis was made. Elevated blood pressure, tachycardia, dyspnea, and mucus hypersecretion occurred as a consequence of the catecholamine crisis after EUS-FNB for paraganglioma. After the diagnosis, the beta-blocker was intended to be replaced with phenoxybenzamine; however, due to the unavailability of this drug in the hospital, terazosin, an alpha-blocker, was used instead. The patient’s condition improved after a week of controlled and adjusted terazosin use, and the patient was transferred to a general ward.

**Figure 5.**
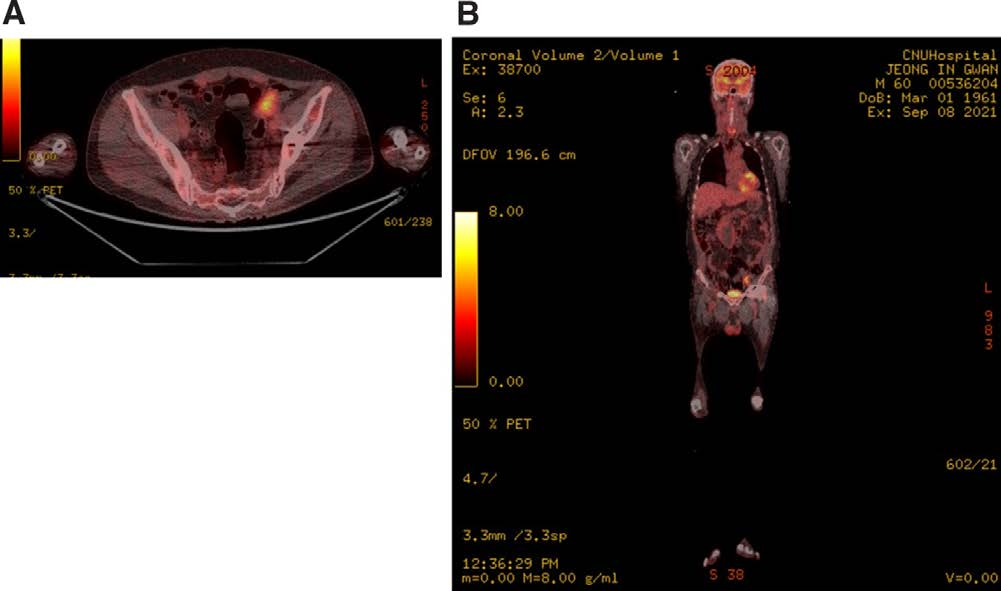
PET-CT for paraganglioma work-up. A hypermetabolic mass is observed in the proximal portion of the sigmoid colon. (5A) Axial plane. (5B) Coronal plane. PET = positron emission tomography.

On positron emission tomography taken for the evaluation of the paraganglioma, increased glucose uptake was further observed in the proximal sigmoid colon, which made it necessary to differentiate paraganglioma from colon cancer. No metastatic findings were found (Fig. 5). On sigmoidoscopy, an ulcero fungating mass was observed, which was narrowing the lumen of the sigmoid colon. The histological evaluation lumen was observed on sigmoidoscopy performed to differentiate sigmoid colon lesions. Histological examination confirmed the presence of adenocarcinoma and not a paraganglioma; thus, the diagnosis of colon cancer was made.

The patient had both masses in the sigmoid colon and retroperitoneum removed at the same time during surgery and is currently being followed up by surgeons and endocrinologists.

## 3. Discussion

Paraganglioma is a rare neoplasm. The combined annual estimated incidence rate of pheochromocytoma and paraganglioma is approximately 0.8 per 100,000 person-years,^[5]^ and in the United States, approximately 500 to 1600 cases are reported annually.^[6]^ Paraganglioma is an extra-adrenal pheochromocytoma originating from chromaffin cells of the sympathetic, parasympathetic, and neural paraganglia.^[7]^ That is, if it originates from chromaffin cells in the adrenal gland, it is called pheochromocytoma, and if it originates from extra-adrenal chromaffin cells, it is called paraganglioma.

Approximately 75% of sympathetic paragangliomas occur in the abdomen, most commonly at the organ of Zuckerkandl, at the junction of the vena cava and the left renal vein, or at the bifurcation of the aorta near the septum of the inferior mesenteric artery. Approximately 10% occur in the thoracic region, including the pericardium.^[8,9]^ Approximately 10% to 15% of these tumors are nonfunctioning, and in the other 10%, there is no clinical manifestation of hormonal activity.^[10]^ Therefore, diagnosis and management in the absence of symptoms such as hypertension, palpitations, headache, and sweating is challenging.

Incision or fine-needle biopsy for catecholamine-producing tumors such as paraganglioma or pheochromocytoma is contraindicated because it may lead to a fatal catecholamine crisis.^[11]^ As seen in this case patient, catecholamine-induced hypertension or hypotension may develop and cardiovascular collapse could occur. Additionally, an increase in circulating catecholamine may lead to stress-induced cardiomyopathy. Acute pulmonary edema in this patient was caused due to increased catecholamine levels. Firstly, an increase in blood catecholamine results in systemic vasoconstriction and an increase in venous return. As a result, plasma oncotic pressure increases and pulmonary edema occurred. Secondly, acute hypertension due to catecholamine causes damage to the contractility of the left ventricle, leading to acute left ventricular failure. As a result, plasma volume increases and pulmonary edema occurs. Pulmonary edema resulting from a catecholamine crisis is called neurogenic pulmonary edema.^[12]^

Our patient was on beta-blocker for heart rate control; however, his uncontrolled blood pressure substantiated sinus tachycardia. Beta-blockers can counteract the vasodilatory effects of beta-2 adrenoreceptors, potentially causing vasoconstriction due to unopposed alpha-adrenoreceptor stimulation and, consequently, hypertensive crisis,^[13]^ which is why it is contraindicated to use beta-blocker before administering alpha-blocker in cases of catecholamine crisis.

When reviewing this case retrospectively, it can be seen that the patient was unintentionally managed only according to contraindications. EUS-FNB is a useful, less-invasive, and safe diagnostic method for obtaining pathological specimens from various types of lesions, particularly pancreatic tumors, and abdominal or mediastinal lymph nodes.^[4]^ However, as in the present case, complications not easily conceived may occur. Therefore, before performing EUS-FNB, patient history taking and physical examination are critical. When performing a biopsy of a mass near the aorta or inferior vena cava, it is advisable to perform the procedure after fully considering the possibility of catecholamine-producing tumor.

## Author contributions

**Conceptualization:** Eaum-Seok Lee.

**Data curation:** Hyun Seok Lee.

**Methodology:** Eaum-Seok Lee.

**Writing – original draft:** Hyun Seok Lee.

**Writing – review & editing:** Eaum-Seok Lee.
